# A modified Susceptible-Infected-Recovered model for observed under-reported incidence data

**DOI:** 10.1371/journal.pone.0263047

**Published:** 2022-02-09

**Authors:** Imelda Trejo, Nicolas W. Hengartner

**Affiliations:** 1 Theoretical Biology and Biophysics Group, Los Alamos National Laboratory, Los Alamos, New Mexico, United States of America; 2 Center for Nonlinear Studies, Los Alamos National Laboratory, Los Alamos, New Mexico, United States of America; International Prevention Research Institute, FRANCE

## Abstract

Fitting Susceptible-Infected-Recovered (SIR) models to incidence data is problematic when not all infected individuals are reported. Assuming an underlying SIR model with general but known distribution for the time to recovery, this paper derives the implied differential-integral equations for observed incidence data when a fixed fraction of newly infected individuals are not observed. The parameters of the resulting system of differential equations are identifiable. Using these differential equations, we develop a stochastic model for the conditional distribution of current disease incidence given the entire past history of reported cases. We estimate the model parameters using Bayesian Markov Chain Monte-Carlo sampling of the posterior distribution. We use our model to estimate the transmission rate and fraction of asymptomatic individuals for the current Coronavirus 2019 outbreak in eight American Countries: the United States of America, Brazil, Mexico, Argentina, Chile, Colombia, Peru, and Panama, from January 2020 to May 2021. Our analysis reveals that the fraction of reported cases varies across all countries. For example, the reported incidence fraction for the United States of America varies from 0.3 to 0.6, while for Brazil it varies from 0.2 to 0.4.

## Introduction

Susceptible-Infected-Recovered (SIR) models, introduced by Kermack and McKendrick and further developed by Wilson and Worcester [1, 2], have been extensively used to describe the temporal dynamics of infectious disease outbreaks [3–5]. They have also been widely used to estimate the disease transmission rate by fitting the models to observed incidence data [6–8], such as time series of daily or weekly reported number of new cases provided by [9–12], for example. Implicit in all these model fittings is the assumption that all the infected individuals have been observed. Yet that assumption is problematic when disease incidences are under-reported. Under-reporting of incidence is prevalent in health surveillance of emerging diseases [13, 14], and also occurs when a disease presents a large fraction of asymptomatic carriers, e.g., Typhoid fever, Hepatitis B, Epstein-Barr virus [15] and Zika [16]. Lack of systematic testing and the presence of sub-clinical patients, which are prevalent in both Severe Acute Respiratory Syndrome Coronavirus 2 (SARS-CoV-2), the causative agent of the coronavirus disease (COVID-19) pandemic [17–20], and Influenza [21, 22], also leads to under-counting incidence and death. Directly fitting an SIR model to raw under-reported incidence will underestimate the transmission rate (see Under-estimation of the transmission rate section). Therefore, failing to account for the under-reporting will under-estimate the severity of the outbreak, possibly leading decision makers to call the epidemic under control prematurely.

To account for under-reporting in an SIR-type model, Shutt *et. al* [ 23] propose to split the infected individuals into two: an observed category and an unobserved category. This is a special case of the Distributed Infection (DI) models introduced in [24]. However, fitting this model to data is problematic since there are no data from the unobserved category. Furthermore, making inferences about DI model parameters is difficult as there are no adequate stochastic model extensions for the DI models, which implies that there is no analytic expression for the likelihood. A partial solution of this problem is to use Approximate Bayesian Computations as in [23] or rely on particle filtering [25]. Finally, we mention two recent approaches to model asymptomatic individuals in SIR-type models: First Lopman *et. al*. in [26] model Norovirus outbreaks using an SEIR model, with E standing for “exposed”, where the infected would progress from symptomatic to asymptomatic to immune. Once immune, individuals could cycle between immune and asymptomatic infection. Second, Kalajdzievska *et. al*. in [15] propose an SIcIR model, with Ic standing for “infectious carrier”, where infected individuals are separated into asymptomatic and symptomatic groups by a given probability as they progress from the susceptible group.

The aim of this paper is to present a novel approach to estimate the under-reported from reported incidence data and apply this methodology to COVID-19 incidences. The COVID-19 pandemic is a particular example of an infectious disease that poses many challenges in quantifying the under-reported incidence, and hence estimating its infectiousness [19, 27, 28], as under-reporting arises from the presence of sub-clinical infections [20, 29], asymptomatic individuals [30, 31], and lack of systematic testing [17, 18]. Accordingly, asymptomatic individuals account for 20–70% of all the infections [30]. Additionally, early in the China outbreak, before traveling restrictions, 86% of all infections were not documented [19].

In the development of our methodology, we present two innovations: First, we introduce an alternative to the DI models that directly describes the dynamics of the observed under-reported incidences. Specifically, assuming that a constant fraction of the newly infected individuals is observed, we derive a set of integral-differential equations describing the local temporal dynamics of the observed incidence. Second, we use the local dynamics of the observed incidence to propose a model for the conditional expectation of new cases, given the observed past history. Making additional distributional assumptions, we obtain a likelihood for the epidemic model parameters: the transmission rate *β*, and the fraction *p* of observed incidence. We refer to Bettencourt and Ribeiro [32] for an interesting alternative framework that leads to a likelihood for the basic reproduction number $R_{0}$. We show that as the epidemic progresses, both of these parameters become identifiable.

## Materials and methods

### Data source

The time series of the daily number of confirmed COVID-19 cases and total population, *N*, of the eight analyzed countries, were obtained from World Health Organization (WHO) reports. Both data sets can be freely downloaded online: https://covid19.who.int/WHO-COVID-19-global-data.csv and https://worldhealthorg.shinyapps.io/covid/, respectively [12, 33]. We used all available incidence reports up to the present study, which corresponds to the reports from January 03, 2020 to May 18, 2021.

### Model development

Our epidemic model is developed in three steps. First, we extend a generalized SIR model to describe the dynamics of the observed (under-counted) infections. Second, we introduce a local version of that SIR model to describel the evolution of the epidemic in a series of observational time windows given the past time serie of observed incidences. This more flexible model is used to compute the conditional expectation of current observed incidence given the past history. Third, we develop a computationally tractable approximation for the conditional expectation to speed up Monte-Carlo Markov Chain (MCMC) inferences of our model parameters.

#### Generalized SIR model

Classical mass-action epidemic models, such as the SIR models, are simple yet useful mathematical descriptions of the temporal dynamics of disease outbreak [3–5]. These models describe the temporal evolution of the number of susceptible *S*(*t*), infected *I*(*t*) and recovered *R*(*t*) individuals in a population of fixed size *N* = *S*(*t*) + *I*(*t*) + *R*(*t*). We model their dynamics through the set of integral-differential equations [34]:

$$\begin{array}{ccc} S^{\prime }\left(t\right) & = & -\frac{\beta }{N}S\left(t\right)I\left(t\right) \end{array}$$ (1)

$$\begin{array}{ccc} I(t) & = & \int_{0}^{t}(-S^{\prime }\left(u\right))\left(1-F\left(t-u\right)\right)du+I\left(0\right)\left(1-F\left(t\right)\right) \end{array}$$ (2)

$$\begin{array}{ccc} R(t) & = & \int_{0}^{t}(-S^{\prime }\left(u\right))F\left(t-u\right)du+R\left(0\right)+I\left(0\right)F\left(t\right), \end{array}$$ (3)

with initial conditions *S*(0), *I*(0), *R*(0). The parameter *β* measures the transmission rate (also called infection rate [35, 36]) and the function *F*(*t*) is the cumulative distribution of the time from infection to recovery. When *F*(*t*) = 1 − *e*^−*γt*^, the exponential distribution with mean *γ*^−1^, our model reduces to the standard SIR model (see Murray [37] for example). For completeness, the proof of existence and uniqueness of the solution of System (1)–(3) is provided in the appendix. An alternative proof can be found in [34].

The model parameters *β* and *F*(*t*) are epidemiologically relevant and provide insights into the outbreak. For example, the basic reproductive number as defined by Lotka [38, 39]:

$$\begin{array}{c} R_{0}=\beta \int_{0}^{\infty }\frac{S(t)}{N}\left(1-F\left(t\right)\right)dt\approx \frac{\beta }{\gamma }. \end{array}$$ (4)

The term *S*(*t*)/*N* in Eq (4) is the fraction of susceptible individuals in the population that can be infected and $\gamma^{-1}=\int_{0}^{\infty }\left(1-F\left(t\right)\right)dt$ is the average recovery time. $R_{0}$ is arguably the most widely used measure of the severity of an outbreak [40, 41], at least in the absence of interventions to control it. It measures the expected number of secondary infections attributed to the index case in a naïve population. Other quantities of interest, such as the maximum number of infected individuals and the total number of infections, can be expressed in terms of the reproductive number $R_{0}$, e.g. Weiss [36].

For many diseases, it is reasonable to assume that the disease progression from infection to recovery is known, either because the disease is well characterized, or because the date of onset of symptoms, hospital admissions, and discharge data are available [42]. Thus we will assume throughout this paper that we know the distribution of the recovery period *F*(*t*) and we will focus on estimating the transmission rate *β*.

#### Modeling the observed disease incidence

Let $\tilde{S}\left(t\right)$, $\tilde{I}\left(t\right)$ and $\tilde{R}\left(t\right)$ denote the observed number of susceptible, infected and recovered individuals as a function of time. We make the following modeling assumptions:

(A1) The true underlying dynamics follows the SIR dynamics described by Eqs (1)–(3) with known fixed population size *N* and time-to-recovery distribution *F*(*t*).
(A2) A constant fraction *p* of newly infected individuals is observed, that is ${\tilde{S}}^{\prime }\left(t\right)=pS^{\prime }\left(t\right)$, with 0 < *p* < 1. The same fraction *p* of initial cases is observed, i.e., $\tilde{I}\left(0\right)=pI\left(0\right)$, $\tilde{R}\left(0\right)=pR\left(0\right)$, and $\tilde{S}\left(0\right)=N-\tilde{I}\left(0\right)-\tilde{R}\left(0\right)$.
(A3) The recovery distribution is the same for observed and unobserved infected individuals.

Under these assumptions, the observed number of infected individuals at time *t* is

$$\begin{array}{c} \tilde{I}\left(t\right)=\int_{0}^{t}(-{\tilde{S}}^{\prime }\left(u\right))\left(1-F\left(t-u\right)\right)du+\tilde{I}\left(0\right)\left(1-F\left(t\right)\right)=pI\left(t\right), \end{array}$$ (5)

and similarly, $\tilde{R}\left(t\right)=pR\left(t\right)$. The number of observed susceptible individuals is

$$\begin{array}{c} \tilde{S}\left(t\right)=\left(1-p\right)N+pS\left(t\right). \end{array}$$ (6)

Eq (6) follows by solving the differential equation ${\tilde{S}}^{\prime }\left(t\right)=pS^{\prime }\left(t\right)$ and using the identity $\tilde{S}\left(0\right)=N\left(1-p\right)+pS\left(0\right)$, which results from (A2) and *N* = *S*(0) + *I*(0) + *R*(0).

These equations capture the intuitive idea that under-reported incidence results in a larger number of observed susceptible and fewer infected and recovered individuals through the epidemic evolution. Consider the ratio, which yields from Assumption (A2) and Eqs (5) and (6):

$$\begin{array}{c} \frac{-{\tilde{S}}^{\prime }\left(t\right)}{\tilde{S}\left(t\right)\tilde{I}\left(t\right)}=\frac{-pS^{\prime }\left(t\right)}{pI\left(t\right)[(1-p)N+pS(t)]}=\frac{\beta }{N}\frac{S(t)}{\left(1-p)N+pS(t\right)}=\frac{\beta }{N}v\left(t\right). \end{array}$$ (7)

For a standard SIR model with *p* = 1, the ratio *v*(*t*) is unity. However, for the observed process, the ratio *v*(*t*) starts at one and then monotonically decreases over time. It follows that fitting an SIR model to observed incidence data, neglecting the under-reporting, will produce a nearly unbiased, but possibly noisy estimate for *β* early in the outbreak when *v*(*t*) ≈ 1. As more data becomes available and *v*(*t*) decreases, the estimated transmission rate *β* will under-estimate the true value. As a consequence, one might at later times in an outbreak underestimate the severity of the outbreak and call the epidemic under control prematurely.

The following theorem describes the dynamics of the observed number of susceptible, infected and recovered individuals when only a fraction *p* of the infected individuals are observed.

**Theorem 1** *Under assumptions (A1), (A2), and (A3), the process of the observed individuals evolves according to the following set of integral-differential equations*:

$$\begin{array}{ccc} {\tilde{S}}^{\prime }\left(t\right) & = & -\frac{\beta }{Np}\tilde{S}\left(t\right)\tilde{I}\left(t\right)+\frac{\beta (1-p)}{p}\tilde{I}\left(t\right) \end{array}$$ (8)

$$\begin{array}{ccc} \tilde{I}\left(t\right) & = & \int_{0}^{t}(-{\tilde{S}}^{\prime }\left(u\right))(1-F(t-u))du+\tilde{I}\left(0\right)(1-F(t)) \end{array}$$ (9)

$$\begin{array}{ccc} \tilde{R}\left(t\right) & = & \int_{0}^{t}(-{\tilde{S}}^{\prime }\left(u\right))F\left(t-u\right)du+\tilde{R}\left(0\right)+\tilde{I}\left(0\right)F\left(t\right). \end{array}$$ (10)

The conclusion of the theorem follows from algebraic manipulations of Eqs (1) to (6). The addition of the positive term $\left(\left(1-p\right)\beta /p\right)\tilde{I}\left(t\right)$ to ${\tilde{S}}^{\prime }\left(t\right)$ implies a slower depletion rate of the observed susceptible population than would be expected under the standard SIR model. Note that this positive term must be small enough such that $-{\tilde{S}}^{\prime }\left(t\right)\geq 0$, for all *t* ≥ 0 and all *p* > 0, condition imposed from Assumption (A2). Assumption (A2) of observing the same fraction *p* of initial infected and recovered individuals was established only for the technical mathematical proofs of Eq (5) and $\tilde{R}\left(t\right)=pR\left(t\right)$. This mathematical assumption will be relaxed in the following local dynamics definition.

#### A stochastic model for the observed incidence

Observed incidences of disease are typically reported at regular time intervals. Precisely, let 0 = *t*_0_ < *t*_1_ < *t*_2_ < … < *t*_*n*_ denote the boundaries of the observation windows. For simplicity, we assume that *t*_*k*_ = *k*Δ, and we denote by *Y*_*k*_ the number of new cases of the disease observed in the interval (*t*_*k*−1_, *t*_*k*_], *k* = 1, 2, …, *n*. We also assume that the new cases *Y*_*k*_ depend on the actual observed past history of incidences $H_{k-1}=\left\{Y_{1},Y_{2},\ldots ,Y_{k-1}\right\}$. As a result, our model takes into account the impact of fluctuations in the reports. Indeed, imagine that the reported cases *Y*_*k*_ are much larger than what is predicted by Model (8)–(10). That excess of cases will alter the observed dynamics of the outbreak, making it progress faster. Similarly, smaller numbers of incidences will slow down the outbreak. The following model takes into account past fluctuations in the incidence to model locally the dynamics of the process at each time interval given the past history.

**Definition 1** *Let Y*_1_, *Y*_2_, …, *Y*_*k*_ *be the sequence of observed incidences and assume that the cumulative probability distribution F for the time to recovery is continuous. We model the local dynamics of the observed number of susceptible* ${\tilde{S}}_{k}\left(t\right)$ *and infected* ${\tilde{I}}_{k}\left(t\right)$ *individuals at time t in the interval* (*t*_*k*−1_, *t*_*k*_] *through the set of differential-integral equations*

$$\begin{array}{ccc} {\tilde{S}}_{k}^{\prime }\left(t\right) & = & -\frac{\beta }{Np}{\tilde{S}}_{k}\left(t\right){\tilde{I}}_{k}\left(t\right)+\frac{\beta (1-p)}{p}{\tilde{I}}_{k}\left(t\right) \end{array}$$ (11)

$$\begin{array}{ccc} {\tilde{I}}_{k}\left(t\right) & = & \int_{t_{k-1}}^{t}\left(-{\tilde{S}}_{k}^{\prime }\left(u\right)\right)(1-F(t-u))du\\ & & \;+\sum_{j=1}^{k-1}\frac{Y_{j}}{\Delta }\int_{t_{j-1}}^{t_{j}}\left(1-F\left(t-u\right)\right)du+\tilde{I}\left(0\right)(1-F(t)), \end{array}$$ (12)

*with initial conditions* ${\tilde{S}}_{k}\left(t_{k-1}\right)=\tilde{S}\left(0\right)-\sum_{j=1}^{k-1}Y_{j}$ *and with the convention that* $\sum_{j=1}^{0}Y_{j}=0$, where both ${\tilde{S}}_{k}\left(t_{k-1}\right)>0$ *and* $-{\tilde{S}}_{k}^{\prime }\left(t\right)\geq 0$ *for all t* ≥ 0 *and p* > 0. *For this model, the conditional expectation of incidence given the past history is*

$$\begin{array}{c} \mu_{k}=E\left[Y_{k}|Y_{1},Y_{2},\ldots ,Y_{k-1}\right]=\int_{t_{k-1}}^{t_{k}}\frac{\beta }{Np}{\tilde{S}}_{k}\left(u\right){\tilde{I}}_{k}\left(u\right)-\frac{\beta (1-p)}{p}{\tilde{I}}_{k}\left(u\right)du, \end{array}$$ (13)

*for all k* = 1, 2, …, *n*.

**Remark 1** *Continuity of the cumulative distribution of the time to recovery F implies that* ${\tilde{I}}_{k}\left(t_{k}\right)$ *is left continuous. Furthermore, if F has a probability density, then* ${\tilde{I}}_{k}\left(t\right)$ *admits a right-hand derivative at t*_*k*−1_.

The local model described in Definition 1 has the same infection dynamics, Eq (11), as the global model. What differs is the evolution of the number of infected individuals, and how it relates to the history of past incidences. The following heuristic serves to motivate Eq (12) in Definition 1. Decompose the integral in Eq (9) for the number of infected individuals into a sum over each observed window (*t*_*j*−1_, *t*_*j*_] to write

$$\begin{array}{ccc} \tilde{I}\left(t\right) & = & \int_{t_{k-1}}^{t}(-{\tilde{S}}^{\prime }\left(u\right))(1-F(t-u))du\\ & & \;+\sum_{j=1}^{k-1}\int_{t_{j-1}}^{t_{j}}(-{\tilde{S}}^{\prime }\left(u\right))(1-F(t-u))du+\tilde{I}\left(0\right)(1-F(t)). \end{array}$$

To get ${\tilde{I}}_{k}\left(t\right)$, replace ${\tilde{S}}^{\prime }\left(t\right)$ by its local instantiation ${\tilde{S}}_{k}^{\prime }\left(t\right)$ on (*t*_*k*−1_, *t*_*k*_] and ${\tilde{S}}^{\prime }\left(t\right)$ by *Y*_*j*_/Δ, the empirical rate of new infections, on the interval (*t*_*j*−1_, *t*_*j*_]. This is interpreted as assuming that the *Y*_*j*_ new infections in the interval (*t*_*j*−1_, *t*_*j*_] occur uniformly in that interval. This allows us to take into account the actual number of observed incidence in each time interval instead of using modeled derived quantities, which provides the needs flexibility for our local epidemic model to better track more complex epidemic dynamics than is possible using a global generalized SIR model.

We use the expression for the conditional expectation of incidences in the interval (*t*_*k*−1_, *t*_*k*_] given the time series of past observed incidences in Definition 1 to model the conditional distribution of *Y*_*k*_ given $H_{k-1}=\left\{Y_{1},Y_{2},\ldots ,Y_{k-1}\right\}$. Specifically, we assume that the conditional distribution of $Y_{k}|H_{k-1}$ is negative binomial

$$\begin{array}{c} Y_{k}|Y_{1},Y_{2},\ldots ,Y_{k-1}∼\text{NegBinom}(\frac{\mu_{k}}{\mu_{k}+r},r). \end{array}$$ (14)

A negative binomial counts the number of success in a sequence of identically and independently Bernoulli with probability of success *p* = *μ*_*k*_/(*μ*_*k*_ + *r*) before *r* failures (with probability 1-*p*) occur, *μ*_*k*_ is the conditional expectation defined in Eq (13). With this parametrization, the conditional expectation and variance are

$$\begin{array}{c} E\left[Y_{k}|H_{k-1}\right]=\mu_{k}\;\text{and}\;V\left[Y_{k}|H_{k-1}\right]=E\left[Y_{k}|H_{k-1}\right]+\frac{\mu_{k}^{2}}{r}, \end{array}$$ (15)

respectively. The shape parameter *r* controls the amount of over dispersion when compared to a Poisson distribution for which $V\left[Y_{k}|H_{k-1}\right]=E\left[Y_{k}|H_{k-1}\right]$. In particular, as the shape parameter *r* grows to infinity, the negative binomial model converges to a Poisson distribution with rate *μ*_*k*_. Thus, the negative binomial distribution allows us to account for the extra-Poisson variability that arises in the data. Other distributions are possible, such as beta negative binomial distribution [43] or the Conway-Maxwell-Poisson distribution [44].

With repeated application of the chain rule, we combine the set of conditional distributions for *Y*_*k*_|*Y*_1_, *Y*_2_, …, *Y*_*k*−1_ into a joint likelihood for the model parameters

$$\begin{array}{ccc} L(\beta ,p,r) & = & \prod_{k=2}^{n}P\left[Y_{k}|Y_{1},Y_{2},\ldots ,Y_{k-1}\right]\times P\left[Y_{1}\right] \end{array}$$ (16)

$$\begin{array}{ccc} & = & \prod_{k=2}^{n}\frac{\Gamma (y_{k}+r)}{\Gamma \left(r\right)\Gamma \left(y_{k}+1\right)}\times {\left(\frac{\mu_{k}}{\mu_{k}+r}\right)}^{y_{k}}{\left(\frac{r}{\mu_{k}+r}\right)}^{r}\times P\left[Y_{1}\right] \end{array}$$ (17)

where Γ denotes the gamma function and *μ*_*k*_ depends only on *β* and *p*. Since, in the model formulation, the distribution of *Y*_1_ does not contain any information about the transmission rate and the fraction of observed cases, the term $P[Y_{1}]$ is dropped from the likelihood.

#### Approximation of the conditional expectation

To reduce the computational burden required to numerically solve the set of differential-integral Eqs (11) and (12), and the ensuing integration in Eq (13) to evaluate the conditional expectation, we propose to approximate the conditional expectation *μ*_*k*_ by linearizing both ${\tilde{S}}_{k}\left(u\right)$ and ${\tilde{I}}_{k}\left(u\right)$ around *t*_*k*−1_ in Eq (13), and integrate the result explicitly. The following lemma encapsulates the resulting approximation.

**Lemma 1** *Assume that the cumulative probability distribution F for the time to recovery has a probability density f*. *The conditional expectation μ*_*k*_ *can be approximated by*

$$\begin{array}{c} \mu_{k}=\text{max}(-\Delta {\tilde{S}}_{k-1}^{\prime }[1+\frac{\Delta }{2}(\frac{{\tilde{I}}_{k-1}^{\prime }}{{\tilde{I}}_{k-1}}-\frac{\beta }{p}\frac{{\tilde{I}}_{k-1}}{N})-\frac{\beta \Delta^{2}}{3p}\frac{{\tilde{I}}_{k-1}^{\prime }}{N}],0), \end{array}$$ (18)

*when* ${\tilde{I}}_{k-1}\neq 0$ *and* ${\tilde{S}}_{k-1}/N\geq \left(1-p\right)$, *and μ*_*k*_ = 0 *otherwise. Here*,

$$\begin{array}{ccc} {\tilde{S}}_{k-1}={\tilde{S}}_{k}\left(t_{k-1}\right) & = & \tilde{S}\left(0\right)-\sum_{j=1}^{k-1}Y_{j} \end{array}$$ (19)

$$\begin{array}{ccc} {\tilde{I}}_{k-1}={\tilde{I}}_{k}\left(t_{k-1}\right) & = & \sum_{j=1}^{k-1}\frac{Y_{j}}{\Delta }\int_{t_{j-1}}^{t_{j}}(1-F(t_{k-1}-u))du+\tilde{I}\left(0\right)(1-F(t_{k-1})) \end{array}$$ (20)

$$\begin{array}{ccc} {\tilde{S}}_{k-1}^{\prime }={\tilde{S}}_{k}^{\prime }\left(t_{k-1}\right) & = & -\frac{\beta }{p}(\frac{{\tilde{S}}_{k-1}}{N}-\left(1-p\right)){\tilde{I}}_{k-1} \end{array}$$ (21)

$$\begin{array}{ccc} {\tilde{I}}_{k-1}^{\prime }={\tilde{I}}_{k}^{\prime }\left(t_{k-1}\right) & = & -{\tilde{S}}_{k-1}^{\prime }-\sum_{j=1}^{k-1}\frac{Y_{j}}{\Delta }(F\left(t_{k-j}\right)-F\left(t_{k-j-1}\right))-\tilde{I}\left(0\right)f\left(t_{k-1}\right), \end{array}$$ (22)

*for all k* = 1, 2, …, *n*.

#### Proof of Lemma 1

Eqs (19)–(22) follow directly from the definition of $\tilde{S}\left(t_{k-1}\right)$ and the evaluation of Eqs (11) and (12) at *t*_*k*−1_. To prove Eq (22), we first take the derivative of ${\tilde{I}}_{k}\left(t\right)$, Eq (12), with respect to *t* and simplify it as follows:

$$\begin{array}{c} {\tilde{I}}_{k}^{\prime }\left(t\right)=-{\tilde{S}}_{k}^{\prime }\left(t\right)-\sum_{j=1}^{k-1}\frac{Y_{j}}{\Delta }\int_{t_{j-1}}^{t_{j}}f\left(t-u\right)du+\int_{t_{k-1}}^{t}{\tilde{S}}_{k}^{\prime }\left(u\right)f\left(t-u\right)du-\tilde{I}\left(0\right)f\left(t\right)\;\\ =-{\tilde{S}}_{k}^{\prime }\left(t\right)-\sum_{j=1}^{k-1}\frac{Y_{j}}{\Delta }\left(F\left(t-t_{j-1}\right)-F\left(t-t_{j}\right)\right)+\int_{t_{k-1}}^{t}{\tilde{S}}_{k}^{\prime }\left(u\right)f\left(t-u\right)du-\tilde{I}\left(0\right)f\left(t\right). \end{array}$$

Then, we evaluate at *t*_*k*−1_ and simplify the resulting equation, using the definition of each *t*_*k*_ = Δ*k*:

$$\begin{array}{ccc} {\tilde{I}}_{k}^{\prime }\left(t_{k-1}\right) & = & -{\tilde{S}}_{k}^{\prime }\left(t_{k-1}\right)-\sum_{j=1}^{k-1}\frac{Y_{j}}{\Delta }\left(F\left(t_{k-1}-t_{j-1}\right)-F\left(t_{k-1}-t_{j}\right)\right)-\tilde{I}\left(0\right)f\left(t_{k-1}\right)\\ & = & -{\tilde{S}}_{k}^{\prime }\left(t_{k-1}\right)-\sum_{j=1}^{k-1}\frac{Y_{j}}{\Delta }\left(F\left(t_{k-j}\right)-F\left(t_{k-j-1}\right)\right)-\tilde{I}\left(0\right)f\left(t_{k-1}\right). \end{array}$$

From the definition, both ${\tilde{S}}_{k-1}$ and ${\tilde{I}}_{k-1}$ are non-negative quantities, and the hypothesis ${\tilde{S}}_{k-1}/N\geq \left(1-p\right)$ implies that $-{\tilde{S}}_{k-1}^{\prime }\geq 0$, for all *p* > 0. Therefore, all these equations are well defined. In the proof of Eq (18), the linear approximation of both ${\tilde{S}}_{k}\left(u\right)$ and ${\tilde{I}}_{k}\left(u\right)$ around *t*_*k*−1_ are:

$$\begin{array}{ccc} {\tilde{S}}_{k}\left(u\right) & \approx & {\tilde{S}}_{k}\left(t_{k-1}\right)+\left(u-t_{k-1}\right){\tilde{S}}_{k}^{\prime }\left(t_{k-1}\right) \end{array}$$ (23)

$$\begin{array}{ccc} {\tilde{I}}_{k}\left(u\right) & \approx & {\tilde{I}}_{k}\left(t_{k-1}\right)+\left(u-t_{k-1}\right){\tilde{I}}_{k}^{\prime }\left(t_{k-1}\right). \end{array}$$ (24)

Substituting these equations in the integrand of Eq (13) and solving it yields:

$$\begin{array}{ccc} \mu_{k} & \approx & \frac{\beta }{Np}(\Delta {\tilde{S}}_{k-1}{\tilde{I}}_{k-1}+\frac{\Delta^{2}}{2}\left({\tilde{S}}_{k-1}^{\prime }{\tilde{I}}_{k-1}+{\tilde{I}}_{k-1}^{\prime }{\tilde{S}}_{k-1}\right)+\frac{\Delta^{3}}{3}{\tilde{I}}_{k-1}^{\prime }{\tilde{S}}_{k-1}^{\prime })\\ & & -\frac{\beta (1-p)}{p}(\Delta {\tilde{I}}_{k-1}+\frac{\Delta^{2}}{2}{\tilde{I}}_{k-1}^{\prime })\\ & = & \Delta (\frac{\beta }{Np}{\tilde{S}}_{k-1}{\tilde{I}}_{k-1}-\frac{\beta (1-p)}{p}{\tilde{I}}_{k-1})+\frac{\beta \Delta^{3}}{3Np}{\tilde{S}}_{k-1}^{\prime }{\tilde{I}}_{k-1}^{\prime }\\ & & +\frac{\Delta^{2}}{2}({\tilde{I}}_{k-1}^{\prime }(\frac{\beta }{Np}{\tilde{S}}_{k-1}-\frac{\beta (1-p)}{p})+\frac{\beta }{Np}{\tilde{S}}_{k-1}^{\prime }{\tilde{I}}_{k-1}). \end{array}$$

When ${\tilde{I}}_{k-1}=0$, from Eqs (20) and (22), ${\tilde{S}}_{k-1}^{\prime }=0$ and ${\tilde{I}}_{k-1}^{\prime }=0$. Then *μ*_*k*_ ≈ 0. When ${\tilde{I}}_{k-1}\neq 0$, using the definition of ${\tilde{S}}_{k-1}^{\prime }$ in the previous equation and simplifying it yields:

$$\begin{array}{ccc} \mu_{k} & \approx & -\Delta {\tilde{S}}_{k-1}^{\prime }+\frac{\beta \Delta^{3}}{3Np}{\tilde{S}}_{k-1}^{\prime }{\tilde{I}}_{k-1}^{\prime }-\frac{\Delta^{2}}{2}(\frac{{\tilde{I}}_{k-1}^{\prime }}{{\tilde{I}}_{k-1}}{\tilde{S}}_{k-1}^{\prime }-\frac{\beta }{Np}{\tilde{S}}_{k-1}^{\prime }{\tilde{I}}_{k-1}), \end{array}$$

where the conclusion of Eq (18) follows.

**Remark 2** *Better approximations for μ*_*k*_ *can be obtained using higher order Taylor expansions for* ${\tilde{S}}_{k}\left(u\right)$ *and* ${\tilde{I}}_{k}\left(u\right)$. *This requires the distribution F of time to recovery to have higher order derivatives*.

#### Identifiability

It is known that the measured growth rates in early SIR outbreaks are insensitive to under-reporting. Indeed, in early outbreaks, *S*(*t*) ≈ *N* and hence *I*′(*t*) ≈ (*β* − *γ*)*I*(*t*). Under Assumption (A2), we have that $\tilde{I}\left(t\right)=pI\left(t\right)$ and ${\tilde{I}}^{\prime }\left(t\right)=pI^{\prime }\left(t\right)$, which imply that

$$\begin{array}{c} \frac{d}{dt}\text{log}\;I\left(t\right)=\frac{d}{dt}\text{log}\;\tilde{I}\left(t\right)=\beta -\gamma . \end{array}$$

It follows that the disease incidence grows exponentially with rate *β* − *γ*, irrespective on the fraction *p* of observed incidence. Hence the transmission rate *β* can be estimated if the recovery rate *γ* is known, but the fraction *p* cannot be estimated at that early stage of the outbreak.

As the outbreak matures and moves away from its early exponential growth phase, it becomes possible to estimate both the transmission rate *β* and the fraction *p* of observed cases. The following theorem provides verifiable conditions for both these parameters to be identifiable.

**Theorem 2** *Set*

$$\begin{array}{ccc} U_{k} & = & \int_{t_{k-1}}^{t_{k}}\frac{{\tilde{S}}_{k}\left(u\right)}{N}{\tilde{I}}_{k}\left(u\right)du \end{array}$$ (25)

$$\begin{array}{ccc} V_{k} & = & \int_{t_{k-1}}^{t_{k}}{\tilde{I}}_{k}\left(u\right)du. \end{array}$$ (26)

*If the vector* (*U*_1_, *U*_2_, …, *U*_*m*_) *and* (*V*_1_, *V*_2_, …, *V*_*m*_) *are linearly independent, then β and p are identifiable*.

The proof of Theorem 2 is found in the appendix.

**Remark 3** *As we note earlier, β and and p are not identifiable in the early stages of an outbreak. This is also evident in Theorem 2: In the early stages, we have that* ${\tilde{S}}_{k}\left(u\right)\approx N$, *so that the vectors* (*U*_1_, …, *U*_*k*_) and (*V*_1_, …, *V*_*k*_) *are essentially co-linear. Later in the outbreak, as S*_*k*_(*u*) *is no longer close to N, both parameters become identifiable*.

### Bayesian parameter estimation

We use the Metropolis-Hastings algorithm to draw Monte-Carlo Markov chain (MCMC) [45] samples from the posterior distribution of the model parameters given the epidemic outbreak data. Our implementation transforms the original parameters Θ = (*β*, *p*, *r*) into $\tilde{\Theta }=\left(\xi ,\eta ,\rho \right)\in R^{3}$, where *ξ* = log(*β*), *η* = log(*p*/(1−*p*)) and *ρ* = log(*r*), and selects proposals from a multivariate Gaussian distribution $N\left(\cdot |{\tilde{\Theta }}_{m}\right)=\text{N}\left({\tilde{\Theta }}_{m},\Sigma \right),$ with mean ${\tilde{\Theta }}_{m}$ and diagonal covariance matrix *Σ* with entries 0.001, 0.01, and 0.01. The results presented in the next section are from 40,000 MCMC samples gathered after 40,000 burn-in iterations when starting from Θ_0_ = (0.5, 0.5, 25). Our implementation used the approximation for *μ*_*k*_ presented in Lemma 1.

Following [46, 47], we model the distribution of time to recovery from COVID-19 as the convolution of a lognormal distribution (with mean = 5.2 and sdlog = 0.662) with a Weibull distribution (with mean = 5 and sd = 1.9). The mean and standard error of the resulting recovery time distribution are 10.27 and 4.32, respectively. We refer the interested reader to [30] for a detailed description of additional disease progression parameters of SARS-CoV-2 infection.

Separate chains were run for the time series of incidence data from each country, using all the data from the date of the first confirmed COVID-19 cases to May 18th, 2021 (see Table 1). The assumption of a constant transmission rate does not hold, as each country implemented various mitigation and control strategies, from national lockdown orders to closing of public meeting places (see Table 1 which shows the date on first implementation of mitigation as reported in [48]). To avoid having to model the change in the transmission rate resulting from the implementation of mitigations, our parameter estimation starts on the first day of intervention as reported in Table 1. We still use the whole time series from the time of first confirmed incidence to estimate the number of infected individuals as defined by Eq (12).

**Table 1:** Metadata of analyzed data sets.

Country	Initial reports	Intervention	Population	*n*	*I*(0)
USA	January 20	March 22	331,002,651	485	5
Brazil	February 26	March 24	212,559,417	448	5
Mexico	February 28	March 23	128,932,753	446	6
Argentina	March 03	March 19	45,195,774	442	5
Chile	March 03	March 24	19,116,201	442	5
Colombia	March 06	March 25	50,882,891	439	5
Peru	March 07	March 16	32,971,854	438	9
Panama	March 10	March 24	4, 314,767	435	5

To reduce the impact of weekly reporting patterns (e.g. fewer cases are reported over the weekend) we apply a moving average of seven days to the raw incidence counts before executing the MCMC algorithm. Finally, the initial conditions ${\tilde{S}}_{0}=N-pI\left(0\right)-pR\left(0\right)$, *R*(0) = 0, ${\tilde{I}}_{0}=pI\left(0\right)$ are set using the reported national population counts and number of initial cases as reported in Table 1.

## Results and discussion

### Analysis of COVID-19 incidence data

**Fig 1:**
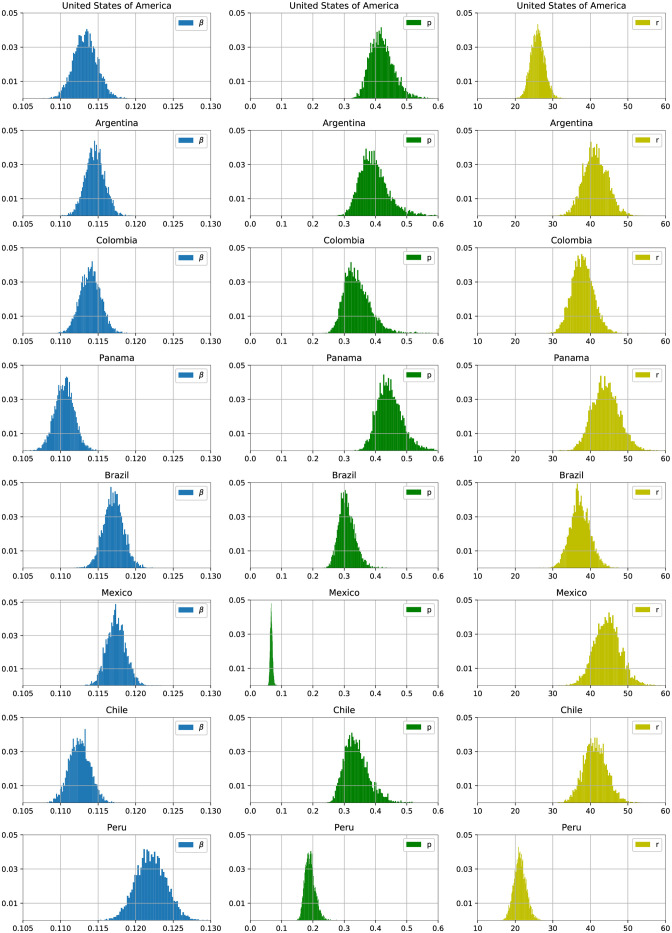
Histograms of the marginal posterior distribution of the transmission rate *β* (left), fraction of reported cases *p* (middle) and the negative binomial shape parameter *r* (right) for each country. The *x*-axis corresponds to the estimated values, and the *y*-axis is the bin’s relative frequency.

We performed separate Bayesian inferences for eight American Countries: the United States of America (USA), Brazil, Mexico, Argentina, Chile, Colombia, Peru, and Panama. Fig 1 shows histograms of the marginal posterior distribution of the transmission rate *β* after the start of mitigation, the fraction observed *p*, and the negative binomial shape parameter *r* for each country. The median and 95% credible intervals of these posterior distributions are presented in Table 2.

**Table 2:** Parameter median values and credible interval estimations.

Country	*β*	95% CI (*β*)	*p*	95% CI (*p*)	*r*	95% CI (*r*)
USA	0.113	[0.110, 0.116]	0.418	[0.359, 0.502]	25.990	[22.728, 29.629]
Brazil	0.117	[0.114, 0.120]	0.307	[0.264, 0.365]	37.133	[32.372, 42.646]
Mexico	0.117	[0.115, 0.120]	0.067	[0.061, 0.076]	44.260	[37.896, 51.527]
Argentina	0.115	[0.112, 0.117]	0.390	[0.324, 0.509]	41.259	[35.451, 47.574]
Chile	0.112	[0.110, 0.115]	0.335	[0.278, 0.431]	41.222	[35.459, 47.478]
Colombia	0.114	[0.111, 0.117]	0.336	[0.277, 0.440]	37.920	[32.705, 43.725]
Peru	0.122	[0.118, 0.126]	0.190	[0.164, 0.226]	21.229	[18.304, 24.525]
Panama	0.110	[0.108, 0.113]	0.443	[0.379, 0.537]	44.003	[37.691, 51.221]

Even though each country used different mitigation strategies, with various level of enforcement, the credible intervals for the transmission parameter of each of the eight countries overlap, with the exception of Peru. There are several hypothesis for why this may be the case: the effectiveness of the various mitigation strategies is compromised by having a small fraction of non-compliant individuals, or most of the benefits of the mitigation strategy are achieved by wearing face masks and moderate social distancing. A third hypothesis is that the estimated transmission rate in our model is a time average of the instantaneous transmission rates, and that averaging lessens the differences in transmission rates.

Similarly, the posterior distributions for the fraction of observed incidence are similar across most of the analyzed countries. The two exceptions are Peru and Mexico, with the under-reporting in Mexico being particularly acute. This is consistent with the observation that Mexico has one of lowest numbers of tests performed per reported case [49]. While an under-reporting factor of about 15 is very large, we believe this effect is real because of how well the model fits the data (see the appendix) and narrowness of the posterior distribution.

Related analyses of COVID-19 data in Mexico have used values for the fraction of reported cases of *p* = 0.2 or *p* = 0.4 to analyze and forecast the evolution of the COVID-19 pandemic and hospital demands [50, 51]. These values are closer to the values that we found for the other Latin American countries. However, these values were not derived from the data. It would be interesting to use our model to investigate the under-reporting in Mexico at a county level to see how the results would differ from local to national levels.

Excess deaths [52] provide an alternative measure of the true impact of COVID-19. Using that measure, [53] reports that COVID-19 deaths in Mexico are under-reported by a factor of 3, whereas we show a factor of 15 for under-reported incidence. This difference may be due differential testing rates of deceased and infected individuals which may arise from the standard of care of severely ill patients admitted to intensive care units that requires COVID-19 testing [50, 54].

Our analysis flags Peru as being different from the other countries both in term of having a higher transmission rate, and a lower reported fraction. Our analysis does not reveal why this is the case, and further analysis incorporating country level explanatory variables to predict transmission rates and under-reporting is needed to uncover the reasons why Peru is different from the other countries in America we studied.

Finally, the estimate of the shape parameter *r* of the negative binomial distribution shows that the relative inflation of the Poisson variance ranges from 2%-5%. That effect is statistically significant. Again, the distributions across the eight countries are commensurate, with the United States and Peru exhibiting more extra Poisson variability than the other countries.

### Under-estimation of the transmission rate

In light of Eq (7), we suggested in the introduction that failing to account for under-reporting leads to underestimating the transmission rate *β*. Here, we numerically demonstrate this effect by fitting an SIR-type model directly to raw incidence data, deliberately neglecting to model under-reporting. The median and 95% credible intervals of the posterior distribution for the transmission rate when modeling under-reporting, and when not are displayed in Table 3.

**Table 3:** Parameter median values and credible interval estimations for the observed *β*_*p*_ and true underlying *β*_1_ rates.

Country	*β*_*p*_	95% CI (*β*_*p*_)	*β*_1_	95% CI (*β*_1_)
USA	0.113	[0.110, 0.116]	0.107	[0.105, 0.109]
Brazil	0.117	[0.114, 0.120]	0.110	[0.108, 0.111]
Mexico	0.117	[0.115, 0.120]	0.105	[0.103, 0.106]
Argentina	0.115	[0.112, 0.117]	0.110	[0.108, 0.112]
Chile	0.112	[0.110, 0.115]	0.107	[0.105, 0.108]
Colombia	0.114	[0.111, 0.117]	0.109	[0.107, 0.110]
Peru	0.122	[0.118, 0.126]	0.109	[0.107, 0.112]
Panama	0.110	[0.108, 0.113]	0.105	[0.103, 0.106]

The parameter *β*_*p*_ coincides with the values from Table 2, while *β*_1_ refers to estimates when the fraction observed is *p* = 1. The posterior distribution for shape parameter *r* were similar for *p* unknown and *p* fixed.

Observe that in all cases, the 95% credible intervals for the transmission rate do not overlap. This shows that knowledge of the fraction of reported incidence is statistically important.

### Variation on the fraction of reported cases

In this section, we consider modeling and estimating a time dependent fraction *p*(*t*) of reported incidence, which can arise from uneven availability of COVID-19 tests [31, 50, 55]. To this end, we model the reported fraction *p*(*t*) with a piece-wise constant function:

$$\begin{array}{c} p\left(t\right)=\sum_{k=1}^{M}p_{k}I_{\left[\xi_{k-1},\xi_{k}\right)}\left(t\right), \end{array}$$ (27)

for all 0 < *t* ≤ *t*_*n*_ ≤ *ξ*_*M*_, where $I_{\left[\xi_{k-1},\xi_{k}\right)}$ denotes the indicator function for each interval [*ξ*_*k*−1_, *ξ*_*k*_). We regularize the sequence of reported fractions *p*_1_, *p*_2_, …, *p*_*M*_ by adding the penalty

$$\begin{array}{c} \frac{M-1}{2}\text{ln}\left(\lambda \right)-\frac{\lambda }{2}\;\sum_{k=2}^{M}{\left(p_{k}-p_{k-1}\right)}^{2}-\frac{M-1}{2}\text{ln}\left(2\pi \right) \end{array}$$ (28)

to the loglikelihood. We assume that the variation between reported fraction, *p*_*k*_ − *p*_*k*−1_, are identically and independently normally distributed with mean zero and variance 1/λ.

**Fig 2:**
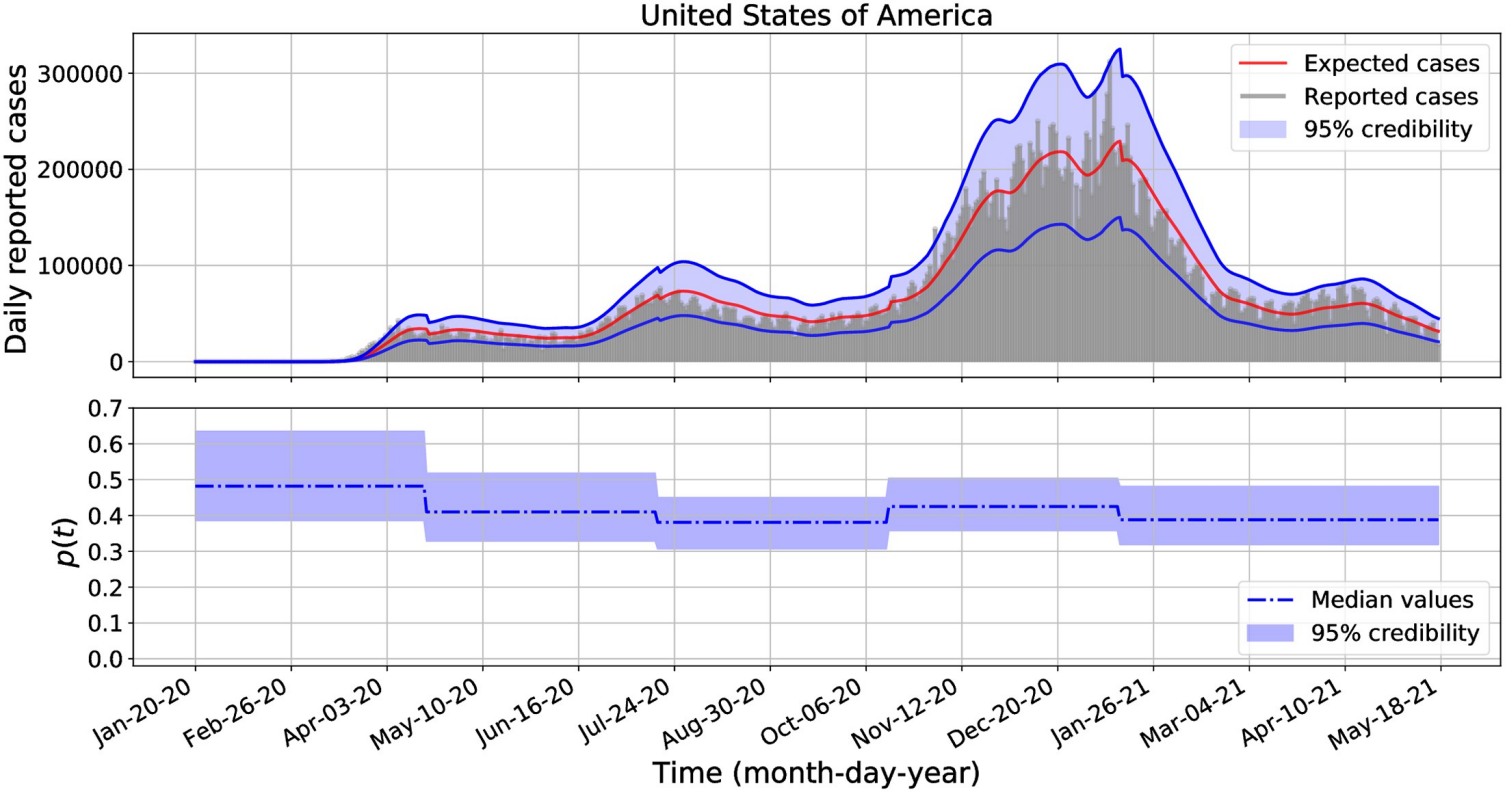
Daily COVID-19 incidence and fraction of reported cases for the United States of America from January 20, 2020 to May 18, 2021.

**Fig 3:**
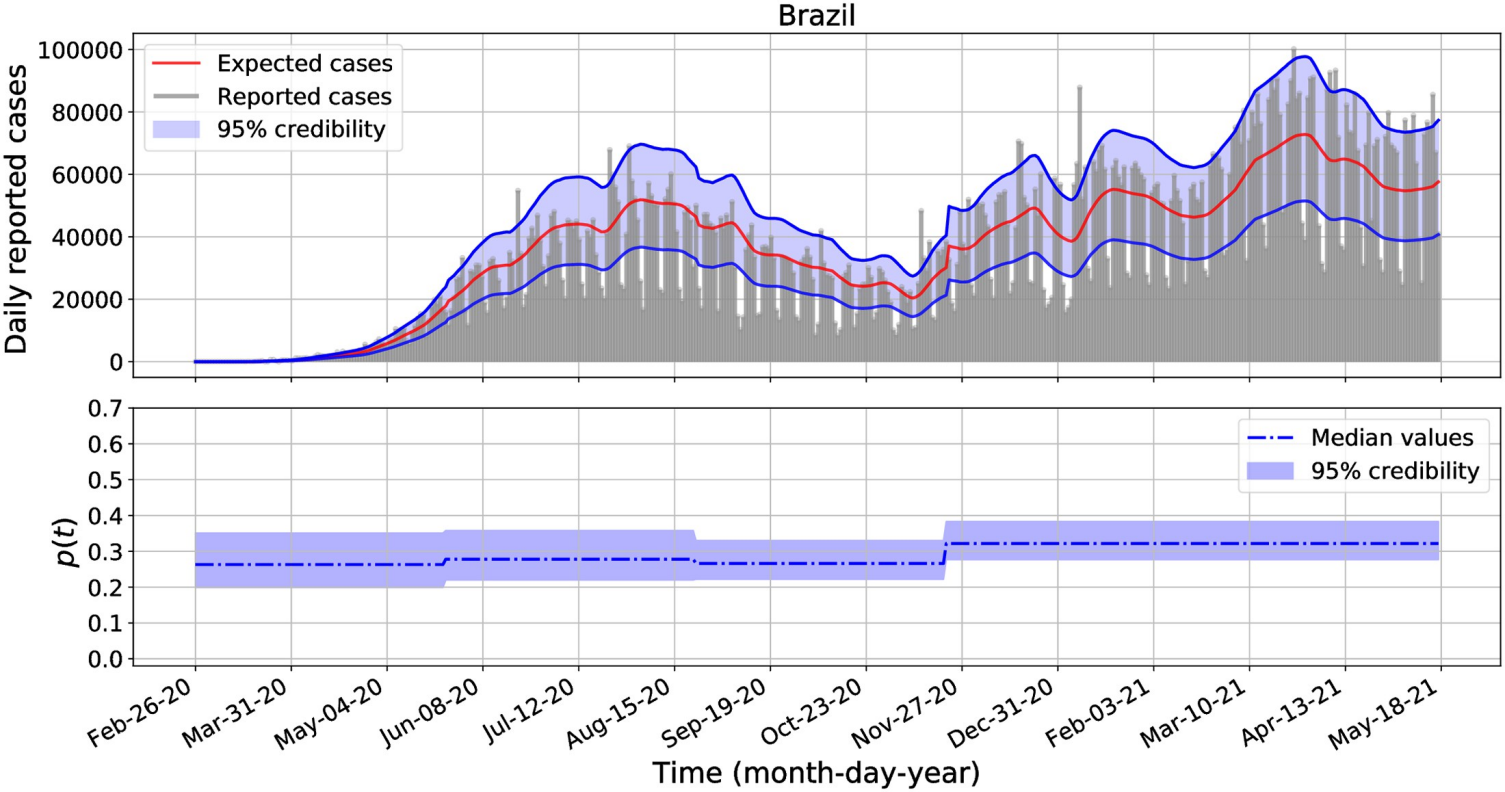
Daily COVID-19 incidence and fraction of reported cases for Brazil from February 26, 2020 to May 18, 2021.

**Fig 4:**
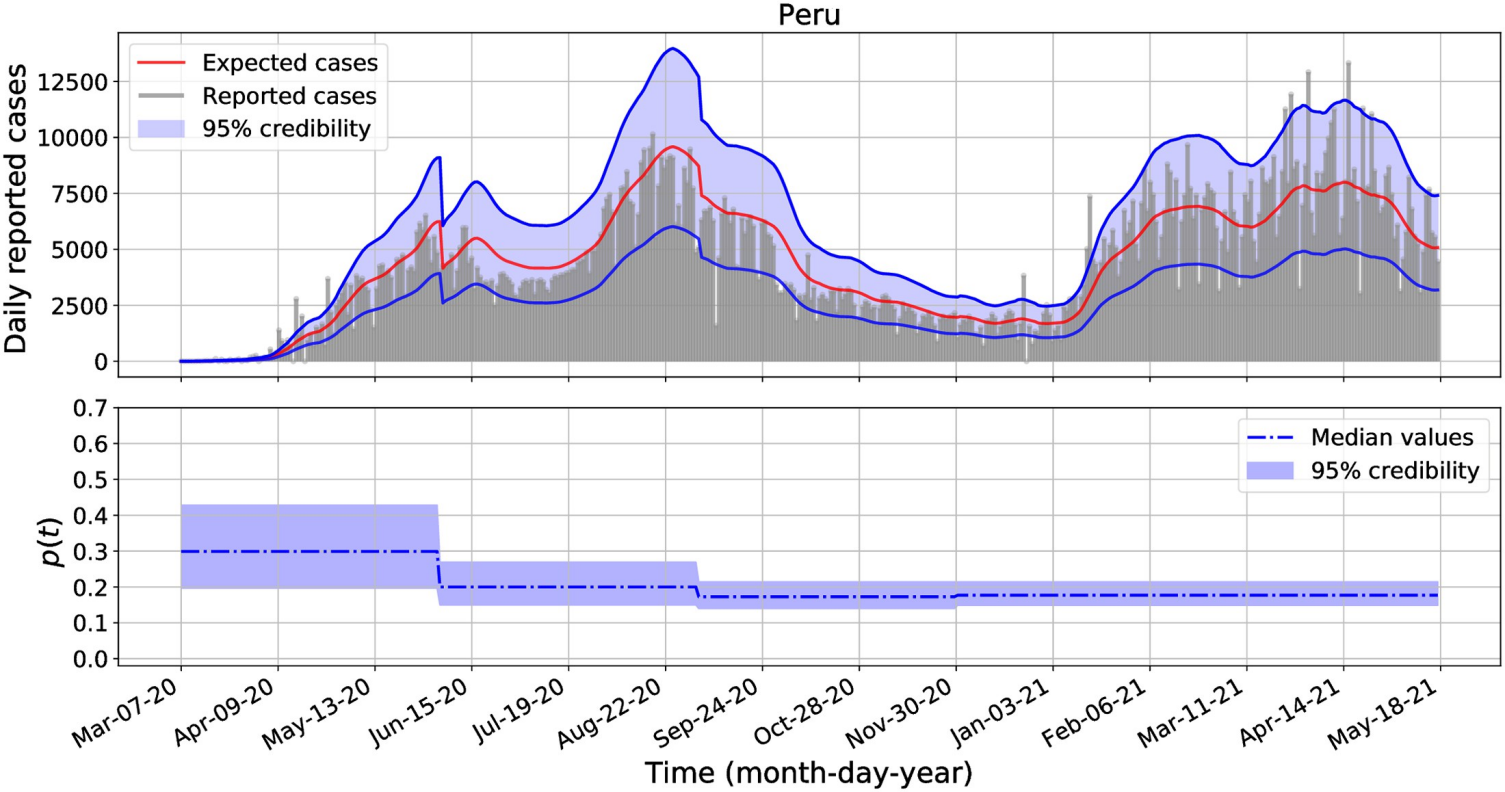
Daily COVID-19 incidence and fraction of reported cases for Peru from March 07, 2020 to May 18, 2021.

Similarly as in the previous section, we performed separate Bayesian inferences to estimate the posterior distributions of *β*, *p*_1_, *p*_2_, …, *p*_*M*_, *r* and λ for each analyzed country: the United States of America, Brazil, Mexico, Argentina, Chile, Colombia, Peru, and Panama. We transform *p*_*k*_ and λ into *η*_*k*_ = log(*p*_*k*_/(1 − *p*_*k*_)) and *l* = log(λ) and use uniform improper priors on the transformed parameters. We defined *p*(*t*) with constant pieces of length modulo 90 days and we use the equations from Lemma 2 to compute the expected incidence *μ*_*k*_. These equations generalize the equations from Lemma 1 when *p* = *p*_*k*_ for all *k* = 1, 2, …, *M*, see the appendix for further details. The median and 95% credible intervals of the posterior distributions of *β*, *r*, and λ are presented in Table 4. For clarity in the presented results for λ values, we decided to round them to the nearest integer values. The analogous results for the posterior distributions for each reported fraction, *p*_1_, *p*_2_, *p*_3_, *p*_4_ and *p*_5_, are plotted in the second panel of Figs 2–4 for the United States of America, Brazil, and Peru. The corresponding results for Mexico, Argentina, Chile, Colombia, and Panama are shown in the second panel of S1–S5 Figs. In all cases, the 95% credible intervals for each *p*_*k*_ values are displayed in the blue-shadow areas, while their median values are plotted in blue-dashed-dotted lines.

**Table 4:** Parameter median values and credible interval estimations varying the *p* values.

Country	*β*	95% CI (*β*)	*r*	95% CI (*r*)	λ	95% CI (λ)
USA	0.114	[0.111, 0.117]	26.259	[22.833, 29.993]	231	[25, 1843]
Brazil	0.118	[0.115, 0.120]	37.876	[32.864, 43.406]	230	[19, 1905]
Mexico	0.119	[0.116, 0.121]	51.400	[44.576, 59.145]	722	[51, 2798]
Argentina	0.114	[0.111, 0.116]	41.818	[35.933, 48.632]	642	[79, 2056]
Chile	0.113	[0.110, 0.116]	41.232	[35.137, 48.253]	786	[86, 2870]
Colombia	0.114	[0.112, 0.117]	38.553	[33.022, 44.653]	364	[18, 2561]
Peru	0.122	[0.118, 0.125]	22.189	[19.138, 25.786]	416	[32, 1262]
Panama	0.110	[0.108, 0.113]	46.731	[39.783, 54.376]	152	[30, 812]

Additionally, in the first panel of Figs 2–4 and S1–S5 Figs we show the credibility of the model estimates for the daily COVID-19 incidence for each country, where the expected median of reported cases, *μ*_*k*_, are plotted in red lines, the upper and lower credible intervals are plotted in blue lines, while the expected incidences lie in the blue-shadow area with probability of 95%. The negative binomial distribution function, Eq (14), was used to build the credible intervals. To estimate the expected cases, the parameter values for *β* and *r* were set equal to the values provided in Table 4 and the *p*_*k*_ values were set to the estimated median values of *p*(*t*) as shown in the second panel of each figure and for each country, respectively.

From Table 4, the marginal posterior distributions for the parameter λ overlap for all analyzed country. All these marginal posterior distributions skewed to the right with large values. For most countries, the credible intervals for *p*(*t*) include a constant function. That is, statistically, we do not have enough evidence to reject the hypothesis that the reported fraction *p*(*t*) for each country is not a constant function during the entire analyzed data set. And for countries that have a small variance λ^−1^ for the increment *p*_*k*_ − *p*_*k*−1_, we have further evidence that *p*(*t*) is nearly constant. The one country for which a constant *p*(*t*) is not retained is Mexico (see S1 Fig).

The second panels of Figs 2–4 shows that there are some variations across all *p*_*k*_ credible intervals for the United States of America, Brazil, and Peru. Interestingly, the credible intervals of *p*_*k*_ for each country are all contained in a wider credible interval than those obtained when assuming a constant fraction *p* of observed cases as reported in Table 2. Comparing Tables 2 and 4, we see that there are not significant changes on the posterior distributions for *β* and *r* when we assume *p* constant and *p* variable. In general, we observe more variation for the observed proportion *p* across the countries than within a country. The latter result is not surprising, as countries implemented different testing policies which may affect the way the incidence data were reported [49, 50, 55].

### Strengths and weaknesses of the proposed local SIR model

Our model locally exploits the SIR dynamics, using past observations to set the initial conditions. This results in a flexible model that can fit complex patterns, such as multiple waves that typically require a time varying transmission rate, with a single parameter. This flexibility comes at a cost: our single estimated transmission rate is a time average of the true time varying one. And while we show that our model empirically fits the data well within the credible intervals, we over-estimate the expected incidence in the valleys and under-estimate near the peaks. It follows that the derived estimates for the reproductive number near a local bottom of an outbreak will have a positive bias, leading to a more conservative view of the effect of mitigation.

Our formulation can be generalized to build epidemic models having non-parametric transmission rates. Such models will alleviate the weakness discussed above, and can be used to identify model-based uncertainties in models. These extensions will be presented in a forthcoming paper. We are also planning to extend the model by incorporating the exposed class, which will provide a more realistic model to study COVID-19 pandemic. As COVID-19 disease progression depends on both the length of time an individual remains in the exposed and infectious classes [30]. This model extension would help us to analyze the effect of different infectious period distributions that could change at the early outbreak due to interventions such as testing, isolation or contact tracing.

Finally, our model has a limited ability to estimate time-varying under-count fractions. Numerical experiments have shown that adding more flexibility to how the latter varies over time degrades our ability to estimate the transmission rate.

## Conclusion

We present a new extension of the standard SIR epidemiological models to study the under-reported incidence of infectious diseases. The new model reveals that fitting a SIR model type directly to raw incidence data will under-estimate the true infectious rate when neglecting under-reported cases. Using the epidemic model we also present a Bayesian methodology to estimate the transmission rate and fraction of under-reported incidence with credible intervals that result directly from incidence data. We also argue that our statistical model can properly track and estimate complex incidence reports, where the resulted estimates update as more data are incorporated.

Using our methodology on the COVID-19 example, we found that the credible intervals for the transmission rates overlap across the eighth analyzed American countries: the United States of America, Brazil, Argentina, Chile, Colombia, Peru, and Panama. In all the cases, the median transmission rates are above 0.105 and below 0.122 (see Tables 2–4). And, for most countries, the credible intervals for the time dependent fraction of reported cases *p*(*t*) include a constant function, and they also provide a range values for the fraction of reported cases per each country. In average, from January 03, 2020 to May 18, 2021: the reported incidence fraction for the United States of America and Panama varies from 0.3 to 0.6; the reported incidence fraction for Brazil, Chile, Colombia, and Argentina varies from 0.2 to 0.5; the reported incidence fraction for Peru varies from 0.15 to 0.35 while for Mexico varies from 0.05 to 0.1 (see Figs 2–4 and S1–S5 Figs).

## Appendix

### Proof of existence and uniqueness of solutions of the generalized SIR model

To prove existence and uniqueness of solutions of System (1)–(3), it is further assumed that the fraction of recovered individuals is defined through a probability distribution function, *F*: [0, ∞) → [0, 1], with the following properties.

**Property 1** *There exists an integrable function f*: [0, ∞) → [0, ∞) *such that*

$$\begin{array}{c} F\left(t\right)=\int_{0}^{t}f\left(u\right)du\;\text{and}\;\int_{0}^{\infty }f\left(u\right)du=1, \end{array}$$

*for all t* ∈ [0, ∞).

**Property 2** *The average recovery time is finite, i.e*.,

$$\begin{array}{c} \frac{1}{\gamma }=\int_{0}^{\infty }\left(1-F\left(t\right)\right)dt<\infty . \end{array}$$

**Theorem 3** *Let U be an open set of* [0, *N*] × [0, *N*] × [0, *N*] × [0, ∞) *and K a compact subset of U containing* (*S*(0), *I*(0), *R*(0), *t*_0_), *the initial condition of System* (1)–(3), *with f*(*t*) *continuously differentiable with respect to t*, *t* ≥ 0 *in U*. *Then there exists a unique solution of System* (1)–(3) *through the point* (*S*(0), *I*(0), *R*(0)) *at t* = 0, *denoted X*(*S*(*t*), *I*(*t*), *R*(*t*), *t*), *with X*(*S*(0), *I*(0), *R*(0), (0)) = (*S*(0), *I*(0), *R*(0)), *for all t such that X*(*S*(*t*), *I*(*t*), *R*(*t*), *t*) ∈ *K*.

**Proof of Theorem 3** From Property 2, System (1)–(3) is well defined and it is equivalent to

$$\begin{array}{ccc} S^{\prime }\left(t\right) & = & -\frac{\beta }{N}S\left(t\right)I\left(t\right)\; \end{array}$$ (29)

$$\begin{array}{ccc} I^{\prime }\left(t\right) & = & \int_{0}^{t}f\left(t-u\right)S^{\prime }\left(u\right)du-S^{\prime }\left(t\right)-I\left(0\right)f\left(t\right) \end{array}$$ (30)

$$\begin{array}{ccc} R^{\prime }\left(t\right) & = & -\int_{0}^{t}f\left(t-u\right)S^{\prime }\left(u\right)du+I\left(0\right)f\left(t\right), \end{array}$$ (31)

which is obtained by taking the derivative with respect to *t* of Eqs (2) and (3) and using Property 1. Therefore, it is enough to prove existence and uniqueness of solutions of System (29)–(31). It follows that the function *G*: *U* → **R**^3^ defined by

$$\begin{array}{c} G\left(S,I,R,t\right)=(S^{\prime }\left(t\right),I^{\prime }\left(t\right),R^{\prime }\left(t\right)) \end{array}$$ (32)

is continuously differentiable in *U*, see for example [56, pp. 32]. Since $\frac{\partial G}{\partial S}$, $\frac{\partial G}{\partial I}$, $\frac{\partial G}{\partial R}$, and $\frac{\partial G}{\partial t}$ exist and are continuous in *U*, then *G* is continuously differentiable in *U*. Therefore, the solution of System (29)–(31) exists for the initial condition *S*(0), *I*(0), *R*(0) and is unique in *K*.

**Proof of Theorem 2** Set *α*_1_ = *β*/*p* and *α*_2_ = *β*(1 − *p*)/*p*. Since

$$\begin{array}{c} p=1-\frac{\alpha_{2}}{\alpha_{1}}\;\text{and}\;\beta =\alpha_{1}-\alpha_{2}, \end{array}$$

identifiability of *α*_1_ and *α*_2_ implies identifiability of *β* and *p*. We can estimate *α*_1_ and *α*_2_ by minimizing the sum of squares

$$\begin{array}{c} \sum_{k=1}^{m}{\left(Y_{k}-\alpha_{1}U_{k}-\alpha_{2}V_{k}\right)}^{2}. \end{array}$$ (33)

The two parameters are identifiable if and only if the vectors (*U*_1_, …, *U*_*m*_) and (*V*_1_, …, *V*_*m*_) are not co-linear.

### Modeling the time dependence fraction of reported incidence

The following definition describes the dynamics of the observed susceptible and infected individuals when constant fractions *p*_*k*_ of infected individuals are observed at each interval (*t*_*k*−1_, *t*_*k*_], i.e., ${\tilde{S}}_{k}^{\prime }\left(t\right)=p_{k}S^{\prime }\left(t\right)$ for all *t* in that interval of time. This hypothesis allows us to study the case when the parameter *p* is a piece-wise time dependent function, as it is defined in Eq (27).

**Definition 2** *Let Y*_1_, *Y*_2_, …, *Y*_*k*_ *be the sequence of observed incidences and assume that the cumulative probability distribution F for the time to recovery is continuous. We model the local dynamics of the observed number of susceptible* ${\tilde{S}}_{k}\left(t\right)$ *and infected* ${\tilde{I}}_{k}\left(t\right)$ *individuals at time t in the interval* (*t*_*k*−1_, *t*_*k*_] *through the set of differential-integral equations*:

$$\begin{array}{ccc} {\tilde{S}}_{k}^{\prime }\left(t\right) & = & -\frac{\beta }{Np_{k}}{\tilde{S}}_{k}\left(t\right){\tilde{I}}_{k}\left(t\right)+\beta {\tilde{I}}_{k}\left(t\right)(\frac{1-p_{k}}{p_{k}}+\sum_{j=1}^{k-1}\frac{Y_{j}\left(p_{k}-p_{j}\right)}{Np_{k}\;p_{j}}+\frac{I\left(0\right)\left(p_{k}-p_{1}\right)}{Np_{k}})\; \end{array}$$ (34)

$$\begin{array}{ccc} {\tilde{I}}_{k}\left(t\right) & = & \int_{t_{k-1}}^{t}(-{\tilde{S}}_{k}^{\prime }\left(u\right))(1-F(t-u))du+p_{k}\sum_{j=1}^{k-1}\frac{Y_{j}}{\Delta p_{j}}\int_{t_{j-1}}^{t_{j}}(1-F(t-u))du\\ & + & p_{k}I\left(0\right)(1-F(t)), \end{array}$$ (35)

*with initial conditions for the observed susceptible individuals*

$$\begin{array}{c} {\tilde{S}}_{k}\left(t_{k-1}\right)=N-p_{1}I\left(0\right)-\sum_{j=1}^{k-1}Y_{j} \end{array}$$ (36)

*and under the hypothesis* 1 ≥ *p*_*k*_ > 0, ${\tilde{S}}_{k}\left(t_{k-1}\right)>0$, *and* $-{\tilde{S}}_{k}^{\prime }\left(t\right)\geq 0$ *for all t and k* = 1, 2, …, *n*. *For this model, the conditional expectation of incidence given the past history is*

$$\begin{array}{c} \mu_{k}=E\left[Y_{k}|Y_{1},Y_{2},\ldots ,Y_{k-1}\right]=\int_{t_{k-1}}^{t_{k}}\left(-{\tilde{S}}_{k}^{\prime }\left(u\right)\right)du, \end{array}$$ (37)

*for all k* = 1, 2, …, *n*.

Note that Definition 1 and Definition 2 are the same when *p* = *p*_*k*_ for all *k* = 1, …, *n*. In the following, we provide the mathematical motivation of Definition 2, using similar ideas as from the derivation of Definition 1.

First, from the definition of *I*(*t*), we re-write Eq (2) as follows:

$$\begin{array}{ccc} I(t) & = & I\left(0\right)(1-F(t))+\sum_{j=1}^{k-1}\frac{1}{p_{j}}\int_{t_{j-1}}^{t_{j}}\left(-p_{j}S^{\prime }\left(u\right)\right)(1-F(t-u))du\\ & + & \frac{1}{p_{k}}\int_{t_{k-1}}^{t}(-p_{k}S^{\prime }\left(u\right))(1-F(t-u))du. \end{array}$$

Similarly for *S*(*t*),

$$\begin{array}{ccc} S(t) & = & S\left(0\right)-\int_{0}^{t}\left(-S^{\prime }\left(u\right)\right)du\\ & = & N-I\left(0\right)-\sum_{j=1}^{k-1}\frac{1}{p_{j}}\int_{t_{j-1}}^{t_{j}}\left(-p_{j}S^{\prime }\left(u\right)\right)du-\frac{1}{p_{k}}\int_{t_{k-1}}^{t}(-p_{k}S^{\prime }\left(u\right))du, \end{array}$$

where in the second equation we used the hypothesis *N* = *S*(0) + *I*(0). Then, from the above two equations, we estimate *I*(*t*) and *S*(*t*) with the equations:

$$\begin{array}{cc} \hat{I}\left(t\right) & =I\left(0\right)(1-F(t))+\sum_{j=1}^{k-1}\frac{Y_{j}}{\Delta p_{j}}\int_{t_{j-1}}^{t_{j}}(1-F(t-u))du\\ & +\frac{1}{p_{k}}\int_{t_{k-1}}^{t}(-{\tilde{S}}_{k}^{\prime }\left(u\right))(1-F(t-u))du \end{array}$$ (38)

$$\begin{array}{ccc} \hat{S}\left(t\right) & = & N-I\left(0\right)-\sum_{j=1}^{k-1}\frac{Y_{j}}{p_{j}}-\frac{1}{p_{k}}\int_{t_{k-1}}^{t}(-{\tilde{S}}_{k}^{\prime }\left(u\right))du, \end{array}$$ (39)

which follow by estimating *p*_*j*_ *S*′(*u*) with ${\tilde{S}}_{j}\left(u\right)$ and then setting ${\tilde{S}}_{j}\left(u\right)=Y_{j}/\Delta$ for all *u* ∈ (*t*_*j*−1_, *t*_*j*_] and all *j* = 1, 2, …, *k*−1. The last equality follows by assuming that the total cases *Y*_*j*_ occur uniformly in the observed interval. Now, solving the integral of Eq (39), with the initial conditions ${\tilde{S}}_{k}\left(t_{k-1}\right)$ defined by Eq (36) and simplifying it, yields:

$$\begin{array}{ccc} \hat{S}\left(t\right) & = & N-I\left(0\right)-\sum_{j=1}^{k-1}\frac{Y_{j}}{p_{j}}-\frac{1}{p_{k}}({\tilde{S}}_{k}\left(t_{k-1}\right)-{\tilde{S}}_{k}\left(t\right))\\ & = & \frac{1}{p_{k}}{\tilde{S}}_{k}\left(t\right)+N(1-\frac{1}{p_{k}})-\sum_{j=1}^{k-1}Y_{j}(\frac{1}{p_{j}}-\frac{1}{p_{k}})-I\left(0\right)(1-\frac{p_{1}}{p_{k}}). \end{array}$$

The above equation implies that ${\tilde{S}}_{k}^{\prime }\left(t\right)=p_{k}{\hat{S}}^{\prime }\left(t\right)$. Therefore, from the estimates $\hat{I}\left(t\right)$ and $\hat{S}\left(t\right)$, Eqs (38) and (39), and the true transmission dynamics process, Eq (1), we have:

$$\begin{array}{ccc} & & \;{\tilde{S}}_{k}^{\prime }\left(t\right)=p_{k}{\hat{S}}^{\prime }\left(t\right)\approx -p_{k}\frac{\beta }{N}\hat{S}\left(t\right)\hat{I}\left(t\right)\\ & & =-\frac{\beta }{N}(\frac{1}{p_{k}}{\tilde{S}}_{k}\left(t\right)+N(1-\frac{1}{p_{k}})-\sum_{j=1}^{k-1}Y_{j}(\frac{1}{p_{j}}-\frac{1}{p_{k}})-I\left(0\right)(1-\frac{p_{1}}{p_{k}})){\tilde{I}}_{k}\left(t\right) \end{array}$$

where ${\tilde{I}}_{k}\left(t\right)=p_{k}\hat{I}\left(t\right)$. Therefore, ${\tilde{S}}_{k}\left(t\right)$ and ${\tilde{I}}_{k}\left(t\right)$ satisfy Definition 2.

The next lemma provides a recursive formula to approximate the conditional expectation *μ*_*k*_ defined by Eq (37). The equation results directly from solving the integral of Eq (37) with the linear approximation of both ${\tilde{S}}_{k}\left(u\right)$ and ${\tilde{I}}_{k}\left(u\right)$ around *t*_*k*−1_. Its proof is similar to the proof of Lemma 1.

**Lemma 2** *Assume that the cumulative probability distribution F for the time to recovery has a probability density f*. *The conditional expectation μ*_*k*_ *can be approximated by*

$$\begin{array}{c} \mu_{k}=\text{max}(-\Delta {\tilde{S}}_{k-1}^{\prime }[1+\frac{\Delta }{2}(\frac{{\tilde{I}}_{k-1}^{\prime }}{{\tilde{I}}_{k-1}}-\frac{\beta }{p_{k}}\frac{{\tilde{I}}_{k-1}}{N})-\frac{\beta \Delta^{2}}{3p_{k}}\frac{{\tilde{I}}_{k-1}^{\prime }}{N}],0), \end{array}$$ (40)

*when* ${\tilde{I}}_{k-1}\neq 0$ *and* $-{\tilde{S}}_{k-1}^{\prime }>0$, *and μ*_*k*_ = 0 *otherwise. Here*,

$$\begin{array}{ccc} {\tilde{S}}_{k-1} & = & N-p_{1}I\left(0\right)-\sum_{j=1}^{k-1}Y_{j} \end{array}$$ (41)

$$\begin{array}{ccc} {\tilde{I}}_{k-1} & = & \frac{p_{k}}{\Delta }\sum_{j=1}^{k-1}\frac{Y_{j}}{p_{j}}\int_{t_{j-1}}^{t_{j}}(1-F(t_{k-1}-u))du+p_{k}I\left(0\right)(1-F(t_{k-1}))\; \end{array}$$ (42)

$$\begin{array}{ccc} {\tilde{S}}_{k-1}^{\prime } & = & -\frac{\beta }{p_{k}}(\frac{{\tilde{S}}_{k-1}}{N}-\left(1-p_{k}\right)-\sum_{j=1}^{k-1}\frac{Y_{j}\left(p_{k}-p_{j}\right)}{Np_{j}}-\frac{I\left(0\right)\left(p_{k}-p_{1}\right)}{N}){\tilde{I}}_{k-1} \end{array}$$ (43)

$$\begin{array}{ccc} {\tilde{I}}_{k-1}^{\prime } & = & -{\tilde{S}}_{k-1}^{\prime }-\frac{p_{k}}{\Delta }\sum_{j=1}^{k-1}\frac{Y_{j}}{p_{j}}(F\left(t_{k-j}\right)-F\left(t_{k-j-1}\right))-p_{k}I\left(0\right)f\left(t_{k-1}\right), \end{array}$$ (44)

*for all k* = 1, 2, …, *n*.

## Supporting information

S1 Fig Daily COVID-19 incidence and fraction of reported cases for Mexico from February 28, 2020 to May 18, 2021. (TIF)

S2 Fig Daily COVID-19 incidence and fraction of reported cases for Argentina from March 03, 2020 to May 18, 2021. (TIF)

S3 Fig Daily COVID-19 incidence and fraction of reported cases for Chile from March 03, 2020 to May 18, 2021. (TIF)

S4 Fig Daily COVID-19 incidence and fraction of reported cases for Colombia from March 06, 2020 to May 18, 2021. (TIF)

S5 Fig Daily COVID-19 incidence and fraction of reported cases for Panama from March 10, 2020 to May 18, 2021. (TIF)
