# Supplementary figures and images for: A modified Susceptible-Infected-Recovered model for observed under-reported incidence data
Source: PLoS One. 2022 Feb 9;17(2):e0263047. doi: 10.1371/journal.pone.0263047 (PMC8827465; doi:10.1371/journal.pone.0263047)

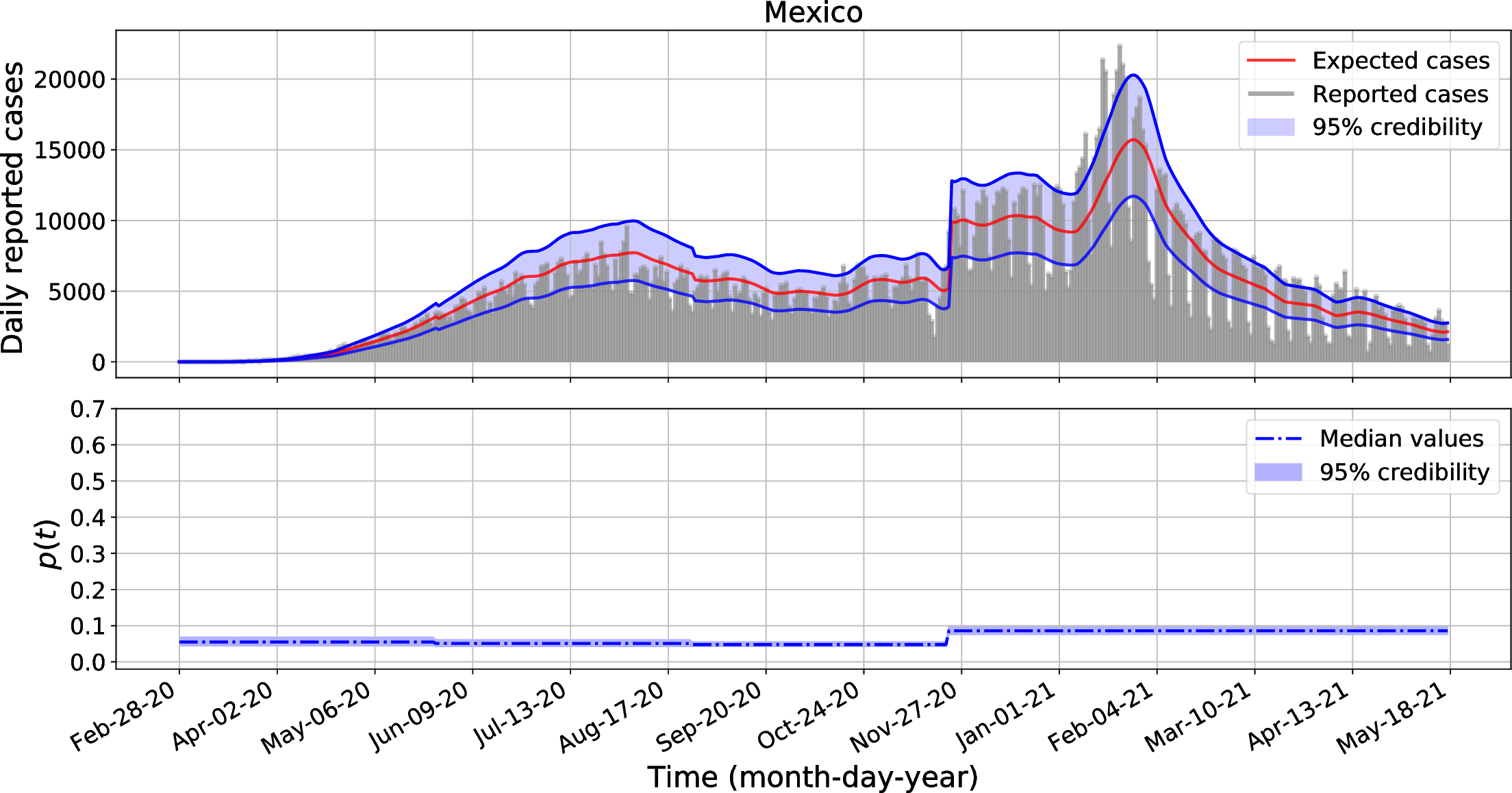

Supplement: S1 Fig — (TIF) [file pone.0263047.s001.tif]

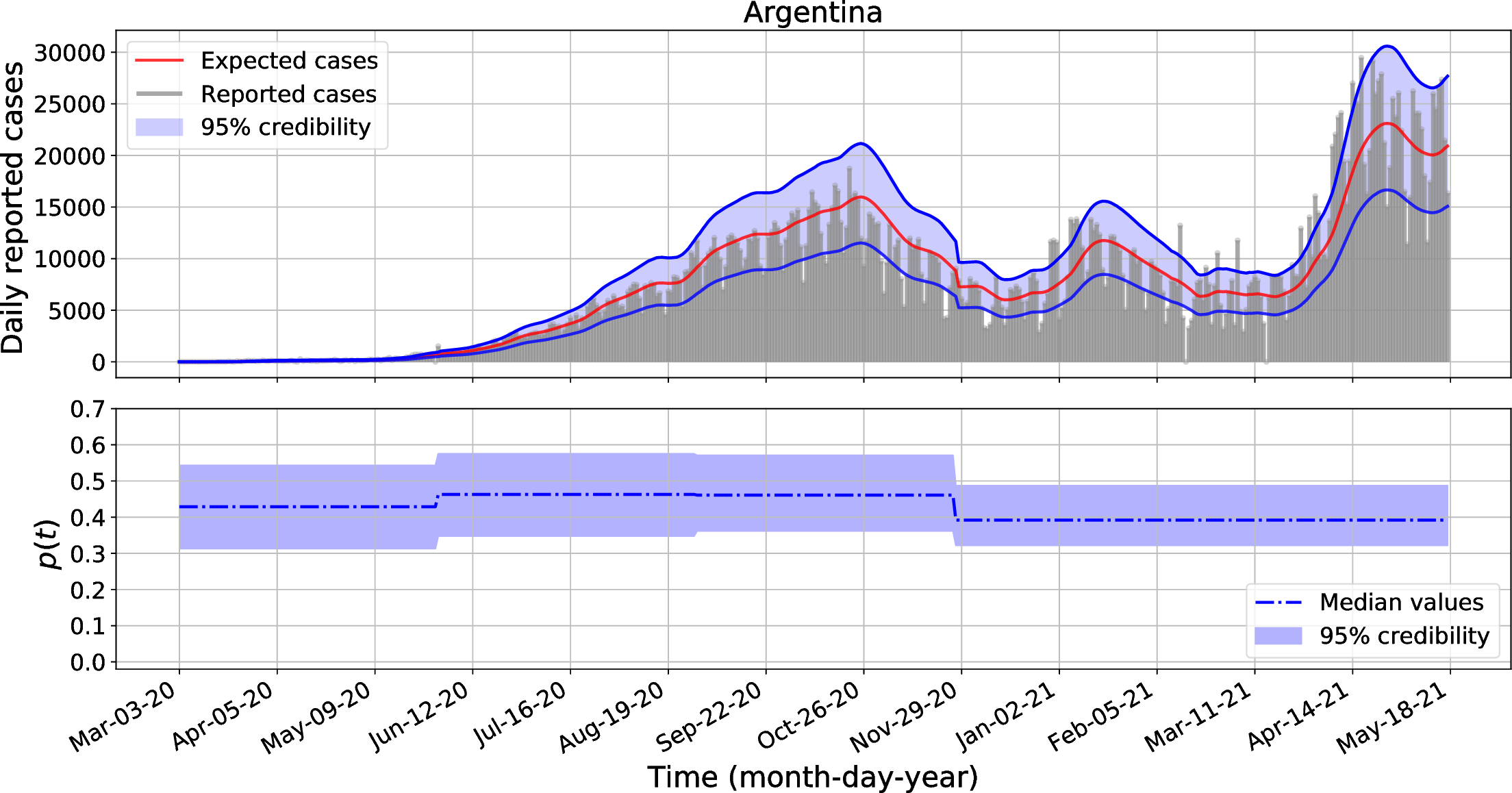

Supplement: S2 Fig — (TIF) [file pone.0263047.s002.tif]

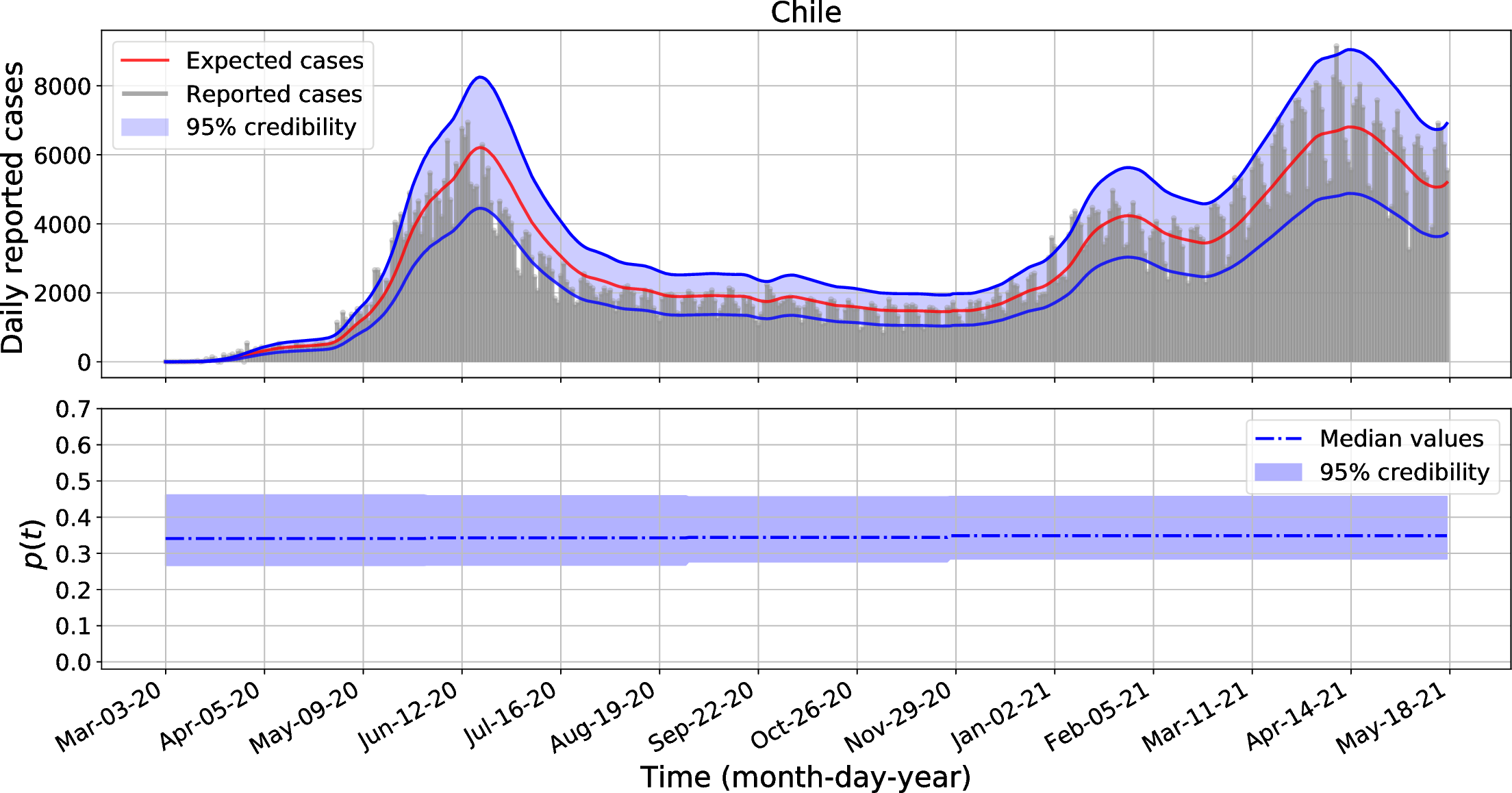

Supplement: S3 Fig — (TIF) [file pone.0263047.s003.tif]

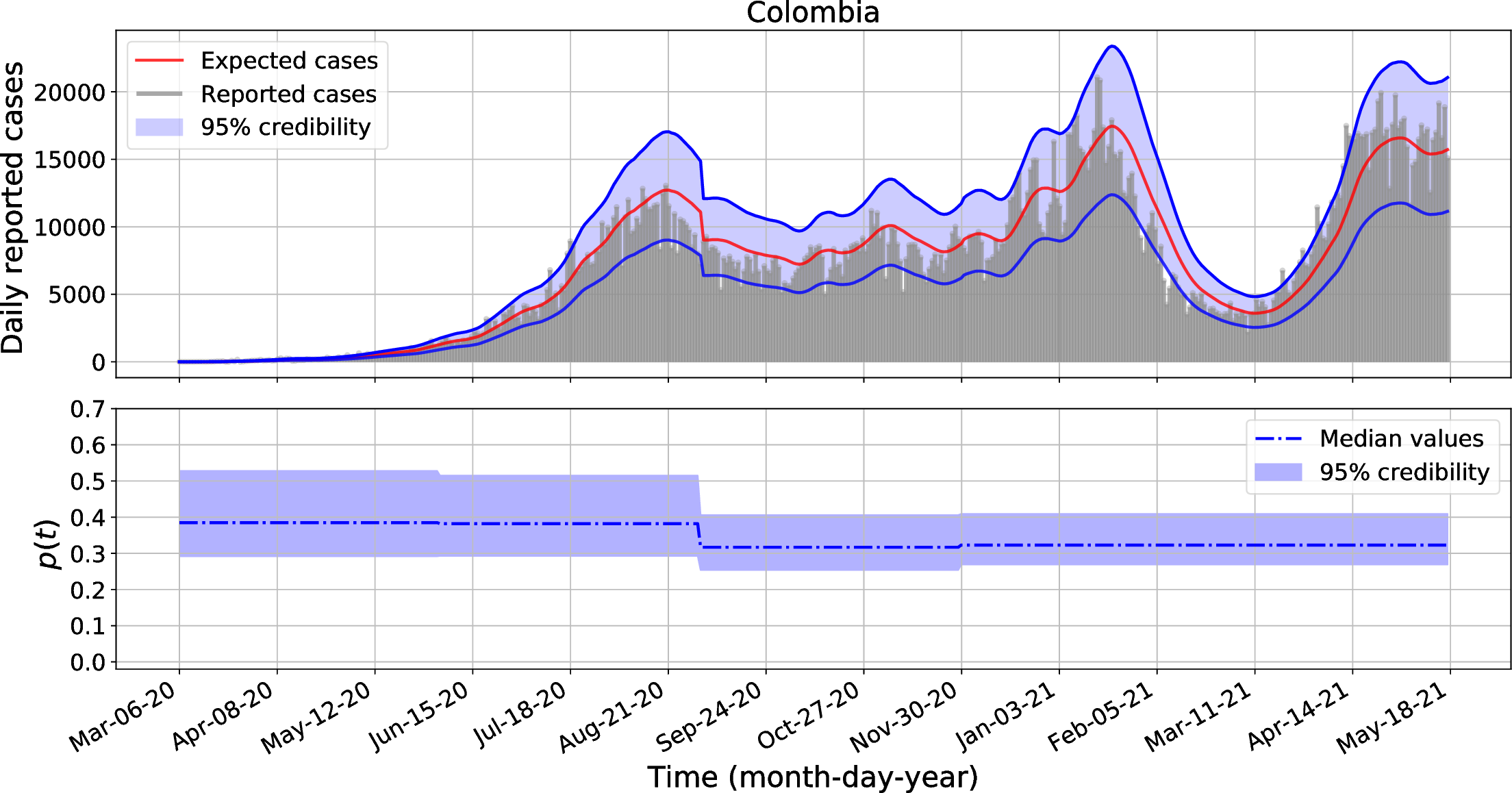

Supplement: S4 Fig — (TIF) [file pone.0263047.s004.tif]

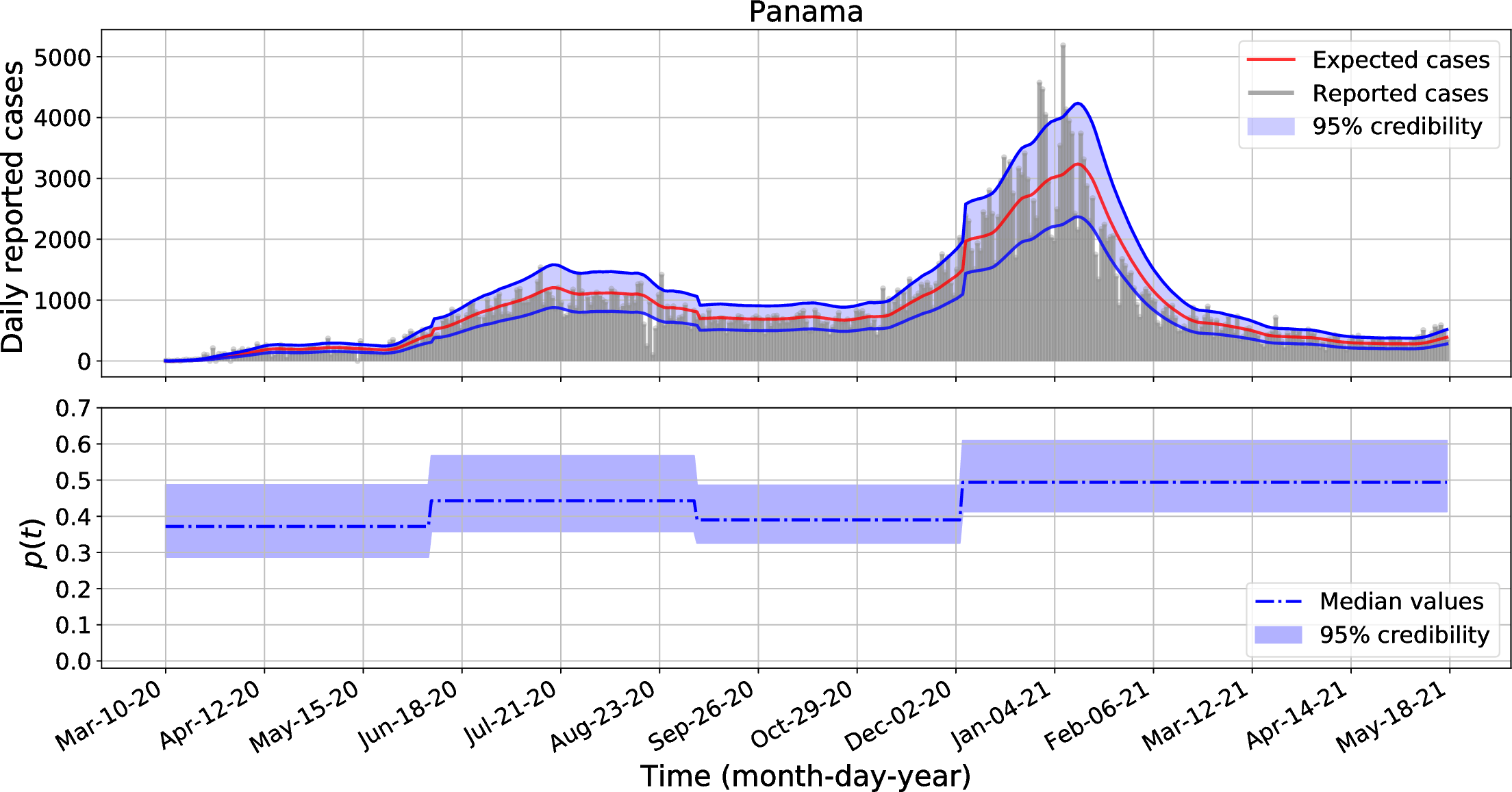

Supplement: S5 Fig — (TIF) [file pone.0263047.s005.tif]
